# Geometric Accuracy Assessment of Deimos-2 Panchromatic Stereo Pairs: Sensor Orientation and Digital Surface Model Production

**DOI:** 10.3390/s20247234

**Published:** 2020-12-18

**Authors:** Manuel A. Aguilar, Rafael Jiménez-Lao, Abderrahim Nemmaoui, Fernando J. Aguilar

**Affiliations:** Department of Engineering, Research Centre CIAIMBITAL, University of Almeria, Carretera de Sacramento s/n, La Cañada de San Urbano, 04120 Almeria, Spain; maguilar@ual.es (M.A.A.); rjl020@ual.es (R.J.-L.); an932@ual.es (A.N.)

**Keywords:** Deimos-2, VHR satellite images, geometric accuracy, sensor orientation model, digital surface model

## Abstract

Accurate elevation data, which can be extracted from very high-resolution (VHR) satellite images, are vital for many engineering and land planning applications. In this way, the main goal of this work is to evaluate the capabilities of VHR Deimos-2 panchromatic stereo pairs to obtain digital surface models (DSM) over different land covers (bare soil, urban and agricultural greenhouse areas). As a step prior to extracting the DSM, different orientation models based on refined rational polynomial coefficients (RPC) and a variable number of very accurate ground control points (GCPs) were tested. The best sensor orientation model for Deimos-2 L1B satellite images was the RPC model refined by a first-order polynomial adjustment (RPC1) supported on 12 accurate and evenly spatially distributed GCPs. Regarding the Deimos-2 based DSM, its completeness and vertical accuracy were compared with those obtained from a WorldView-2 panchromatic stereo pair by using exactly the same methodology and semiglobal matching (SGM) algorithm. The Deimos-2 showed worse completeness values (about 6% worse) and vertical accuracy results (RMSE_Z_ 42.4% worse) than those computed from WorldView-2 imagery over the three land covers tested, although only urban areas yielded statistically significant differences (*p* < 0.05).

## 1. Introduction

The new generation of very high-resolution (VHR) commercial satellites with ground sample distance (GSD) lower than 1 m started in September 1999 with the launching of the IKONOS-2 satellite. Nowadays, an increasing number of VHR optical satellites with stereo (or triplet) image capabilities are commercially available. Among them, we can highlight QuickBird-2 (from 2001 to 2015, 0.61 m GSD), EROS B1 (from 2006, 0.70 m GSD), Resurs-DK1 (from 2006 to 2016, 0.70 m GSD), KOMPSAT-2/Arirang-2 (from 2006, 1 m GSD), IRS Cartosat-2 (from 2007, 0.80 m GSD), WorldView-1 (from 2007, 0.50 m GSD), GeoEye-1 (from 2008 to 2018, 0.41 m GSD), WorldView-2 (from 2009, 0.46 m GSD), Pléiades-HR 1A (from 2011, 0.7 m GSD), Kompsat-3 (from 2012, 0.70 m GSD), Pléiades-HR 1B (from 2012, 0.7 m GSD), Skysat (from 2013, 0.90 m GSD), Kazeosat-1 (from 2014, 1 m GSD), Deimos-2 (from 2014, 1 m GSD), WorldView-3 (from 2014, 0.31 m GSD), Kompsat-3A (from 2015, 0.55 m GSD), TripleSat (from 2015, 1 m GSD), Teleos-1 (from 2015, 1 m GSD), WorldView-4 (from 2016 to 2019, 0.25 m GSD), Vision-1 (from 2018, 0.9 m GSD) and SuperView1A/1B (from 2018, 0.5 m GSD).

Most of these VHR sensors have already been studied over the last 20 years [1,2,3,4,5,6,7,8,9,10,11], with orthoimages and digital surface models (DSM) being the flagship georeferenced products derived from them. Both products need a sensor orientation phase (also known as the triangulation process) as a previous and crucial step. Regarding this sensor orientation phase, a zero-order (rational polynomial coefficient (RPC0)) polynomial transformation is usually the best option when using the triangulation method developed by Grodecki and Dial [12] based on 3D rational functions with vendor image support data (i.e., rational polynomial coefficients (RPC)) refined by a polynomial transformation in the image space. In this case, at least one ground control point (GCP) is necessary to compute the shift parameters through *x* and *y* axes. In fact, RPC0 was the best sensor model for IKONOS-2 [1], GeoEye-1 stereo pairs [4] and GeoEye-1 Geo and WorldView-2 Ortho Ready Standard [7]. However, there are a few VHR satellites (e.g., QuickBird-2) that need a more complex transformation to correct higher order distortions in the images [13]. Due to this, first-order, also called affine transformation (RPC1), and second-order polynomial transformation (RPC2) are also usually applied at the sensor orientation phase.

This work is focused on Deimos-2 sensor, a VHR optical satellite operated by Deimos Imaging that launched on 19 June 2014. There are only a few published works dealing with pansharpening methods [14] or image classification [15] from Deimos-2 imagery. Regarding the geometric capabilities of the Deimos-2 level 1B (L1B) product, a technical report was published by the Joint Research Centre (JRC) in the frame of control with the remote sensing program of the European Common Agriculture Policy [16]. In this work, Vajsová et al. [16] used ground points (GPs) with variable accuracies (from 0.05 m to 0.90 m measured as root mean square error in one direction, RMSE_1D_). Probably due to this poor GP support, they did not find differences between RPC0 and RPC1 sensor models when orthorectifying Deimos-2 images. It is noteworthy that they only tested both sensor models using three and four GCPs.

With regard to DSM generation from VHR satellite stereo pairs, the semiglobal matching (SGM) algorithm [17] is often the most popular in routine production workflows where robustness and efficiency are required. This digital matching algorithm is considered very robust and is able to reduce large outliers in low or non-textured areas while preserving edges and sharp object boundaries [18], being the choice in most of the most recent published works [11,18,19,20]. In Aguilar et al. [19] and Nemmaoui et al. [20], the image matching method based on hierarchical SGM yielded better results than the traditional image matching method based on area-based matching and the cross-correlation threshold. Han et al. [21] tested five commercial and open-source software packages (ERDAS IMAGINE, PCI Geomatica, RSP, MicMac and FORSAT) using a WorldView-1 stereo pair with 0.5 m GSD taken on Terrassa, Spain, in the framework of the ISPRS satellite stereo matching benchmark [22]. In this experiment, RSP (RPC stereo processor by Qin [23]) and PCI Geomatica (PCI Geomatics) were the software packages that best matched the LiDAR DSM benchmark, closely followed by ERDAS IMAGINE and MicMac, while the worst results were attained using FORSAT. It is important to note that RSP, PCI Geomatica, MicMac and ERDAS IMAGINE adopt a SGM implementation [17], while FORSAT [24] applies a multi-feature matching method.

There are several works about DSM generation from VHR satellite imagery over different types of land covers, including urban areas [18,25], agricultural greenhouse areas [20], active tectonics [26], forest [24], glaciers [27] and even small isolated objects [11]. DSM quality assessment is usually carried out by studying its completeness and vertical accuracy [18,19,20,21,25,28], defining completeness as the percentage of correctly matched points over the area of interest [28]. Most of the published works have used an accurate airborne LiDAR-derived DSM as reference [18,19,20,21,29,30,31,32]. To the best of the authors’ knowledge, there are no published works about the extraction of DSM from Deimos-2 L1B stereo imagery.

The aim of this paper is to assess the geopositioning accuracy capabilities of Deimos-2 L1B panchromatic (PAN) stereo imagery for extracting DSM over three different land covers such as plastic covered agricultural greenhouse areas, bare soil, and urban areas. As a previous step, the accuracy of the sensor orientation phase is also statistically analysed based on two sources of variation: (i) sensor orientation model and (ii) number of well-distributed GCPs used in the triangulation process. Once the best combination of sensor orientation model and number of GCPs are selected, the completeness and vertical accuracy of two DSMs photogrammetrically derived from two Deimos-2 stereo pairs over different land covers are computed. Another stereo pair from the well-known WorldView-2 optical sensor taken over the same study area was used as a benchmark for testing the capabilities of Deimos-2 satellite imagery.

The rest of this paper is organized as follows. The study area is described in Section 2. In Section 3, the datasets are described, while Section 4 outlines a detailed explanation of the methodological approach devised to carry out sensor orientation, DSM extraction and accuracy assessment. The results are presented in Section 5 and they are discussed in Section 6. Finally, conclusions are provided in Section 7.

## 2. Study Area

**Figure 1:**
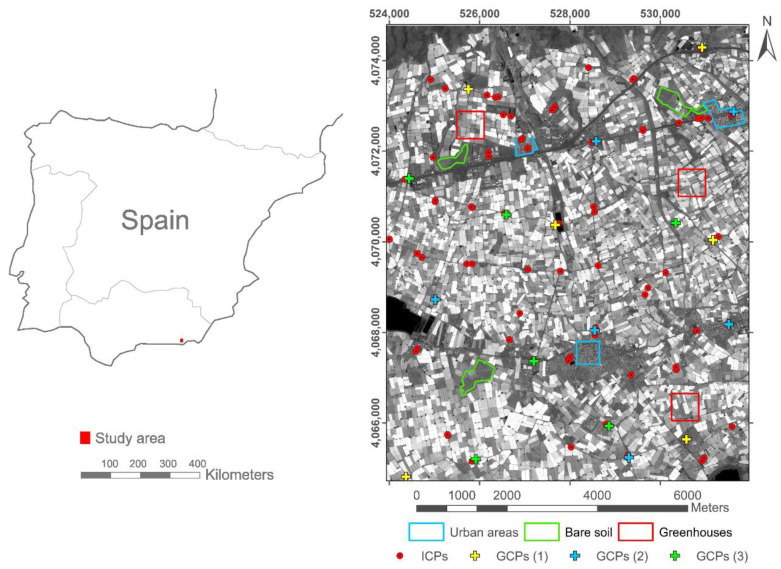
Location of the study area in Almeria (Spain). The nine selected subareas representing urban areas, bare soil and plastic covered greenhouses are depicted in blue, green and red polygons, respectively. Independent check points (ICPs) are represented with red points, while ground control points (GCPs) appear as crosses (six in green, six in blue and six in yellow). Coordinate system: WGS84 UTM zone 30N.

The study area, located in the province of Almeria (Southern Spain), comprises an area of 8000 ha centred on the geographic coordinates (WGS84) 36.7824° N and 2.6867° W (Figure 1). This area is at the core of the greatest concentration of greenhouses in the world [33]. Within the study area, nine sub-plots (blue, green and red polygons in Figure 1) with areas ranging from 14 ha to 36 ha were delineated according to their type of land cover. In fact, three test areas of each land cover were selected so that they were representatives of urban areas, bare soil (practically without vegetation) and plastic covered greenhouses. This study site with the same subareas was already used by Aguilar et al. [19], presenting a smooth relief ranging from 152.6 to 214.8 m above mean sea level.

## 3. Datasets

### 3.1. Ground Points Collection

The three-dimensional coordinates (WGS84 UTM coordinates) of the 102 GPs shown in Figure 1 were obtained by means of a total GPS Topcon HiPer PRO station working in real time kinematic mode and using the satellite’s carrier. The goal was to obtain a reliable measurement of the GPs surveyed providing a position accuracy of better than 10 cm. All these GPs were well-defined and evenly distributed across the whole study area (Figure 1). The entire set of GPs was divided into three sets of six GCPs each to compute the sensor orientation model, leaving the remaining 84 GPs as independent check points (ICPs) to carry out the accuracy assessment (Figure 1).

### 3.2. Deimos-2 Stereo Pairs

Deimos-2 is a VHR optical satellite fully owned and operated by Deimos Imaging, an UrtheCast company. It was launched in 2009 and operates from a Sun-synchronous orbit at a mean altitude of 620 km, which allows an average revisit time of two days worldwide (one day at mid-latitudes). It can provide 1 m PAN and 4 m multispectral (MS) images (R, G, B, NIR) within a swath of 12 km at nadir. The images are distributed in two different processing levels. The level 1B (L1B) product involves calibration and radiometric correction, but it does not include resampling to a map grid. This basic product includes the RPCs (sensor camera model) and the metadata with gain and bias values for each band. The level 1C (L1C) product turns out to be a more elaborated product that has been calibrated, radiometrically corrected, manually orthorectified and resampled to a map grid.

**Figure 2:**
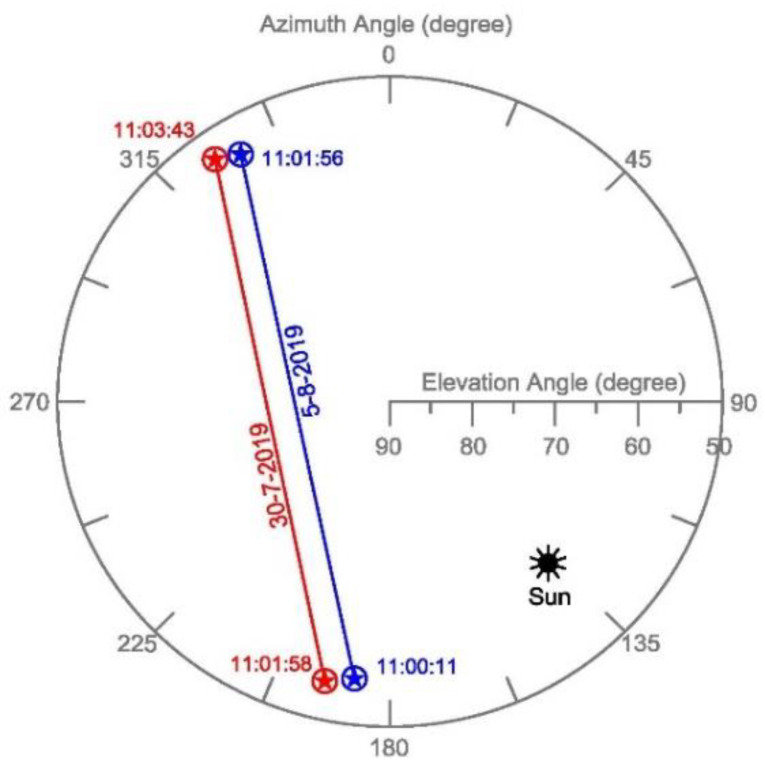
Sky plot with satellite azimuth and elevation angles at the time of acquisition for the two Deimos-2 stereo pairs.

In this study, two Deimos-2 PAN stereo images L1B taken on 30 July 2019 and 5 August 2019 were acquired at the study site. Both stereo pairs had a fairly similar acquisition geometry (Figure 2). The metadata of the four individual PAN Deimos-2 images from the two stereo pairs, including viewing geometry, sun position and other acquisition parameters, are shown in Table 1.

Satellite imaging stereo geometry, measured as convergence angle [34], plays a significant role in the final DSM vertical accuracy [35,36]. The stereo pairs from Deimos-2 used in this work presented similar convergence angles of 64.7 and 65.3 degrees for the first (July) and second (August) acquisition dates, respectively.

### 3.3. WorldView-2 Stereo Pair

WorldView-2 (WV2), launched in October 2009, was the first VHR 8-band MS commercial satellite. This satellite provides 0.46 m GSD PAN and 1.85 m GSD MS, resampled for commercial purpose to 0.5 m and 2 m GSD, respectively [37]. The images are distributed as a basic product or projected onto a plane with a constant height [38]. This second product is known as the Ortho Ready Standard Level 2A product (ORS2A). In this study, a WV2 PAN along-track stereo pair covering the study site was acquired on 5 July 2015. This stereo pair has already been used by Aguilar et al. [19] and Nemmaoui et al. [20]. It was collected in Stereo ORS2A format and also counts on the corresponding RPC sensor camera model and metadata file. The delivered products were ordered with a dynamic range of 11 bits (without dynamic range adjustment) and 0.5 m GSD (PAN). The off-nadir angle for the two images that made up the stereo pair turned out to be 12.6° and 24.6°. The WV2 stereo pair presented a convergence angle of 31.6°.

### 3.4. Reference LiDAR Data

The LiDAR data used as reference in this study were the same as the data used by Aguilar et al. [19]. They were a point cloud in LAS binary file format v. 1.2 [39] collected on 23 September 2015 with orthometric elevations using the EGM2008 geoid. The registered point density of the test area was 0.97 points/m^2^ considering the four returns. The nominal horizontal (RMSE_2D_) and vertical (RMSE_Z_) accuracies had values lower than 0.3 m and 0.2 m, respectively [40].

The LiDAR data for the nine selected subareas were carefully edited by manually removing the points that did not belong to the DSM [19]. Finally, a spatially oriented data thinning was carried out by sub-sampling the edited point cloud using a minimum distance between points of 2 m. This LiDAR was finally used for satellite-based DSM validation. It is important to note that the number of points in the LiDAR data after processing ranged from 20,588 to 58,909 for each of the nine selected subareas shown in Figure 1. The greenhouse subareas (GH1, GH2 and GH3) presented 58,909, 51,920 and 56,000 points, respectively. The urban subareas (UR1, UR2 and UR3) presented 46,677, 50,679 and 25,794 points, respectively, while the bare soil subareas (BS1, BS2 and BS3) had 45,087, 20,588 and 42,815 points, respectively.

## 4. Methods

### 4.1. Sensor Orientation

The four Deimos-2 L1B images of the two stereo pairs were processed individually in the context of the sensor orientation phase. The photogrammetric module of PCI Geomatica v. 2018 (PCI Geomatics, Richmond Hill, Ontario, Canada) called OrthoEngine^®^ was the software used at this stage. Three strategies were studied: (i) direct geopositioning, (ii) all GPs are considered GCPs, and (iii) operational approach by only including a few GCPs.

(i) Direct geopositioning was performed without using GCPs, thus taking the 102 GPs as ICPs in the corresponding four independent projects created in OrthoEngine. In this way, the images were orientated only using the supplied RPCs. Geometric accuracies were computed from the residuals attained at the 102 ICPs and presented as mean error (bias), standard deviation (SD) and RMSE, computed both through *X* and *Y* axes, and as planimetric values (2D).
(ii) The all GPs as GCPs strategy meant using all 102 GPs as GCPs (i.e., without ICPs). Transformations of zero (RPC0), first (RPC1) and second (RPC2) order polynomial adjustments were applied. Geometric accuracies were calculated using the residuals from those 102 GCPs.
(iii) Regarding the operational approach with a few GCPs, three sets of GCPs were chosen to carry out the georeferencing: 6 GCPs (set GCP 1 in Figure 1), 12 GCPs (set GCP 1 plus set 2 in Figure 1), and 18 GCPs (set GCP 1 plus set 2 plus set 3 in Figure 1). Each of these variations of the number of GCPs were tested with the three sensor models mentioned above (i.e., RPC0, RPC1 and RPC2). In total, 36 RMSE_2D_ values were computed (i.e., four single Deimos-2 images × three sets of GCPs (6, 12 and 18) × three sensor models (RPC0, RPC1 and RPC2)). In each case, the remaining GPs were considered ICPs (i.e., 96 ICPs when using 6 GCPs, 90 ICPs when using 12 GCPs, and 84 ICPs when using 18 GCPs) to calculate the corresponding geometric accuracies (bias, SD and RMSE).

All the residual populations through *X* and *Y* axes were tested for normality. The Kolmogorov–Smirnov test was used as an indicator of goodness of fit to a standard normal distribution. Furthermore, no outliers were identified in the residual populations by applying the widely known three-sigma rule [41].

In order to study the influence of the two studied factors (sensor model (i.e., RPC0, RPC1, RPC2) and number of GCPs (no. GCPs)), together with their cross-interactions during the bundle adjustment phase regarding sensor orientation accuracy, an analysis of variance (ANOVA) test was carried out. The observed variable for the designed factorial model with four repetitions [42] was the planimetric RMSE_2D_ computed at ICPs. Regarding this point, it is worth noting that, given such a high number of ICPs, the estimated error (reliability) for RMSE_2D_ calculation could reach values lower than 10% [43]. When the results of the ANOVA test turned out to be significant (*p* < 0.05), the separation of means was carried out using Duncan’s multiple range test at 95% confidence level.

### 4.2. DSM Extraction

As has been already stated, we decided to use exactly the same subareas, reference LiDAR data and methodology as previously used by Aguilar et al. [19] in order to allow the readers to compare the results obtained with different sensors, matching algorithms, software packages and land covers.

In the current work, three stereo pairs (two from Deimos-2 and one from WV2) were used independently to extract the DSM in the study area. The software used was again OrthoEngine (PCI Geomatica v. 2018). OrthoEngine implements a hierarchical SGM approach based on the widely known algorithm proposed by Hirschmüller [17] to generate the disparity maps after applying an epipolar rectification process to the original stereo images.

After the sensor orientations for Deimos-2, following the best operational approach provided by the results described in Section 5.1, and after the WV2 stereo pairs were carried out, three grid spacing format DSMs for each subarea were extracted (two DSMs corresponding to the two Deimos-2 stereo pairs and one DSM corresponding to the WV2 stereo pair). All the DSMs were extracted in orthometric elevations using the EGM2008 geoid without filling blanks (no interpolation) and setting the grid spacing to 1 m [19,21]. In short, 27 unfilled DSMs were extracted corresponding to the nine subareas and the three stereo pairs.

According to several works [18,19,20,21], the quality of the extracted DSMs was assessed by computing their completeness and vertical accuracy.

The completeness of every DSM was computed for the nine studied subareas as the ratio between the number of correctly matched points for the stereo-photogrammetrically extracted DSM and the maximum possible number of points corresponding to the selected DSM grid spacing.

The vertical accuracy assessment of the DSMs derived from the Deimos-2 and WV2 stereo pairs was carried out by using the 3D points from the benchmark LiDAR DSM (see Section 3.4) as ICPs, computing vertical residual (z-residual) at each corresponding point by subtracting the LiDAR height from the photogrammetrically derived height. Note that each ICP will produce a z-residual only if the photogrammetrically derived DSM presents height information in the area around the planimetric position of this ICP.

The exploratory data analysis included standard accuracy statistical measures such as mean, standard deviation (SD), RMSE_Z_ and 95th percentile linear error (LE95). These statistics were computed to assess the final vertical accuracy after removing outliers from the z-residuals populations by applying the three-sigma rule [41]. In addition, and due to the likely presence of outliers in the measurements taken from the original dataset, an additional robust statistic such as normalized median absolute deviation (NMAD) [44] was computed over the residuals. NMAD is a measure of scale or variability that can be considered a consistent estimator for the estimation of standard deviation, also offering the advantage of being very insensitive to the presence of outliers. Note that the three-sigma rule was not applied in the case of the NMAD calculation.

For studying the statistical influence of the two studied factors (i.e., sensor and land cover) on DSM quality, an experimental design based on a factorial model with three samples was used. The Kruskal–Wallis H test [45], a well-known rank-based non-parametric test, was applied to analyse statistically significant differences (*p* < 0.05) between two or more groups of an independent factor in relation to a quantitative dependent variable (mean, SD, RMSE_Z_, LE95, NMAD and completeness). It is important to note that, the residual populations (z-residuals at ICPs) did not always fit a normal distribution, and therefore the ANOVA test could not be used.

## 5. Results

### 5.1. Sensor Orientation

The accuracy results of the direct geopositioning approach computed in object space for each of the four Deimos-2 PAN images are shown in Table 2. Overall, *Y*(North) error was higher than *X*(East) error. The RMSE_2D_ values ranged between 22.9 m and 26.1 m, mainly due to the presence of large systematic errors (bias). The errors in terms of SD (random errors) were around one meter when they were calculated in one dimension, while they ranged from 1.27 m to 1.52 m in planimetry (2D). These values of SD can be considered the best possible geopositioning accuracy results when applying only a simple shift (zero order transformation, RPC0).

Table 3 shows the results of the geometric accuracy for each of the four Deimos-2 images using the RPC0, RPC1 and RPC2 sensor models and the “all GPs as GCPs” strategy (i.e., 102 GCPs). The results were very consistent for all the single images, showing that RPC0 was the worst sensor model for Deimos-2 with a mean RMSE_2D_ value of 1.399 m, presenting statistically significant differences (*p* < 0.05) with respect to both RPC1 and RPC2 sensor models. It is important to note that the mean values of RMSE_2D_ for RPC1 (1.087 m) and RPC2 (1.040 m) sensor models yielded very similar results, not presenting statistically significant differences at a significance level of *p* < 0.05.

The geopositioning accuracy results depending on the number of GCPs (no. GCPs) and sensor model for each of the four Deimos-2 single images are depicted in Table 4. Residuals were measured in the remaining available GPs (i.e., taken as ICPs) in each case. The ANOVA test showed that the applied sensor model presented a significant influence (*p* < 0.05) on the variance model. The other sources of variation, no. GCPs and the cross-interactions between sensor model and no. GCPs, were not statistically significant.

The mean values of the twelve RMSE_2D_ values obtained for each of the three sensor models tested are shown in the first column of Table 4 (values in brackets). The worst statistically significant accuracy results were provided by the RPC0 sensor model, presenting a superscript letter “a” different from the superscript letter “b” of RPC1 and RPC2. The same letter “b” in RPC1 and RPC2 indicates that there are no statistical differences between these two sensor models (*p* < 0.05). The RMSE_2D_ mean values computed with 6, 12 and 18 GCPs were somewhat worse than the results depicted in Table 3 using 102 GCPs, especially for the RPC1 and RPC2 models. The variable no. GCPs obviously influenced the attained results. In fact, a decreasing trend in the RMSE_2D_ values was observed for the RPC1 and RPC2 models as the number of GCPs increased. However, the differences related to the no. GCPs did not result in being statistically significant at a significance level *p* < 0.05. Nevertheless, and by analysing the results shown in Table 4, six GCPs were not enough to obtain robust results from the RPC1 and RPC2 sensor models. Therefore, we recommend using RPC1 with 12 GCPs for carrying out Deimos-2 L1B images’ sensor orientation. For this case, a RMSE_2D_ value of 1.255 m (for the four repetitions corresponding to the four single images) was found, also recording a low SD value of 0.10 m that aims to obtain reasonably stable results.

### 5.2. Quality Assessment of the Extracted DSMs

In view of the results attained in Section 5.1, the sensor model finally applied to both Deimos-2 stereo pairs was the RPC1 model using 12 GCPs (GCPs (1) and GCPs (2) in Figure 1) evenly distributed over the work area. For the WV2 stereo pair, and according to Aguilar et al. [7], the RPC0 model and the same 12 GCPs were used for the orientation phase.

The completeness results obtained from the different DSMs computed in this work are presented in Table 5. From the global statistical analysis of the completeness values using the Kruskal–Wallis H test, it can be concluded that only the land cover factor turned out to be significant (*p* < 0.05). In this sense, the land cover factor presented a partial eta-squared statistic (η_p_^2^) of 70.98%, meaning that 70.98% of the completeness variance was statistically explained by this source of variation. The best completeness was achieved for bare soil, followed by plastic covered greenhouse and, finally, urban areas.

As can be seen in Table 5, no significant differences (*p* < 0.05) were found for plastic covered greenhouse areas between the three stereo pairs used. In the case of urban areas, WV2 yielded the best completeness value, presenting significant differences (*p* < 0.05) with the second stereo pair of Deimos 2 (August 2019) but not with the first one (July 2019). Finally, and regarding bare soil land cover, we found similar results to those observed in urban areas, but only with applying a lower significance level of *p* < 0.10 (Table 5).

**Figure 3:**
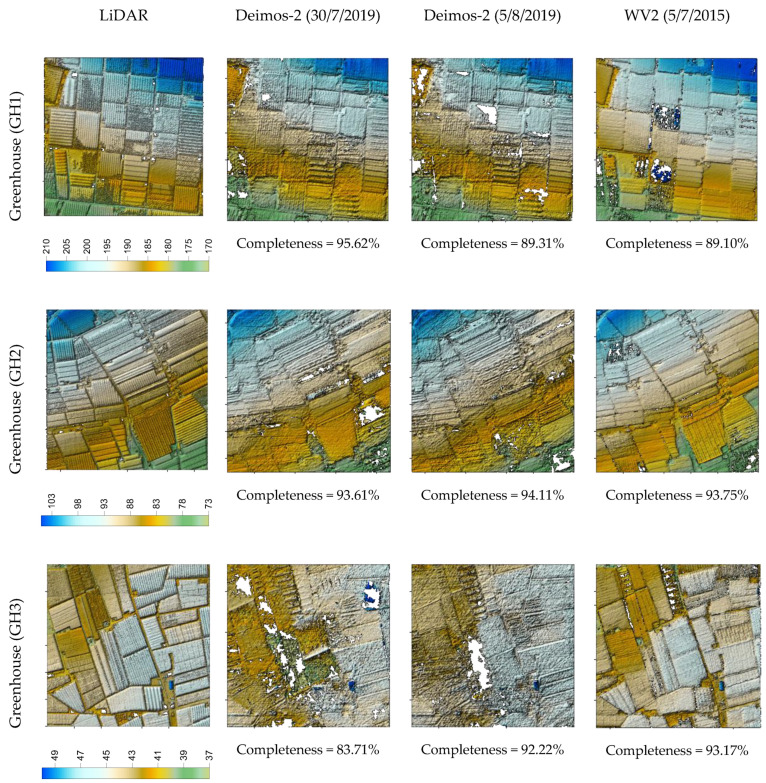
Digital surface models (DSMs) and completeness values corresponding to the three subareas of plastic covered greenhouse land cover (GH1, GH2 and GH3) generated from the first and second Deimos-2 stereo pairs (second and third column) and the WV2 stereo pair (fourth column). The reference LiDAR is depicted in the first column.

**Figure 4:**
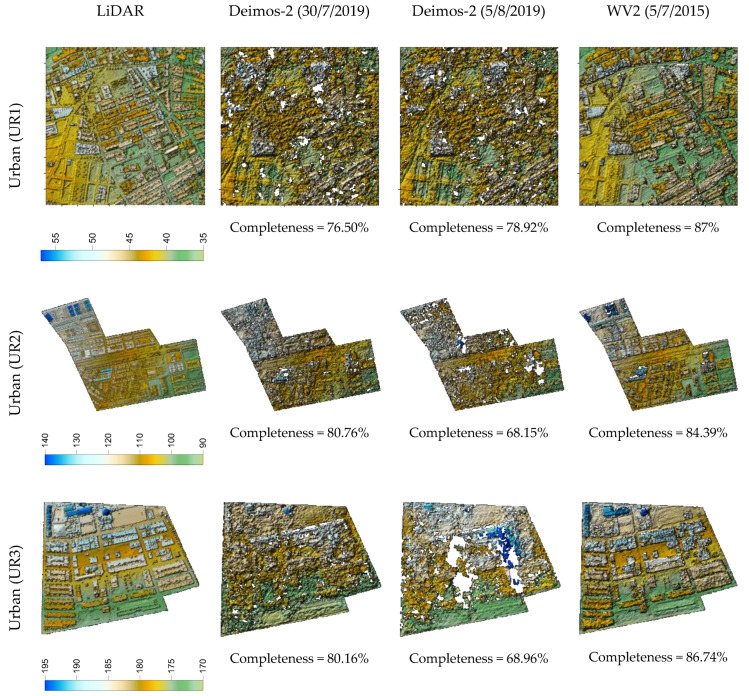
DSMs and completeness values corresponding to the three subareas of urban land cover (UR1, UR2 and UR3) generated from the first and second Deimos-2 stereo pairs (second and third column) and the WV2 stereo pair (fourth column). The reference LiDAR is depicted in the first column.

**Figure 5:**
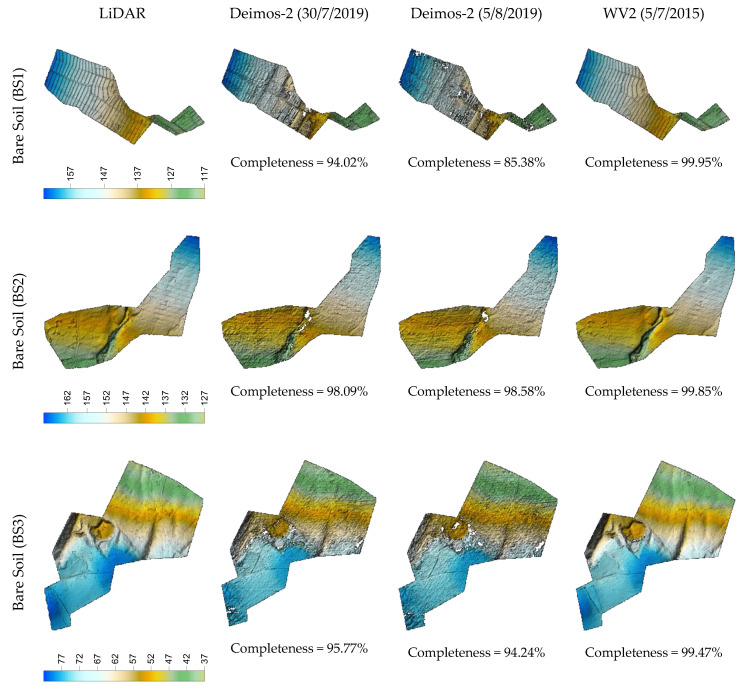
DSMs and completeness values corresponding to the three subareas of bare soil land cover (BS1, BS2 and BS3) generated from the first and second Deimos-2 stereo pairs (second and third column) and the WV2 stereo pair (fourth column). The reference LiDAR is depicted in the first column.

In Figure 3, Figure 4 and Figure 5, we can visually compare the DSMs derived from the two Deimos-2 and WV2 stereo pairs corresponding to plastic covered greenhouse, urban, and bare soil areas, respectively. In each figure, the three subareas per land cover are depicted, together with their corresponding reference LiDAR. Overall, these figures show that DSM completeness was quite similar for Deimos-2 and WV2 in plastic covered greenhouse areas (Figure 3), being slightly better in the case of WV2 in bare soil (Figure 5) and, especially, in urban areas (Figure 4). It can also be observed that Deimos-2′s DSMs were rougher in appearance than WV2′s DSMs (Figure 3 and Figure 5).

Table 6 shows the vertical accuracy results, in terms of mean, SD, RMSE_Z_, LE95 and NMAD, attained for the different DSMs produced in this work. The influence of two factors such as land cover and stereo pair on the descriptive statistics of DSM vertical accuracy is statistically analysed. From a global statistical analysis of the RMSE_Z_ values using the Kruskal–Wallis H test, it can be concluded that the land cover factor turned out to be significant (*p* < 0.05), while the stereo pair source of variation only showed significant differences at a significance level of *p* < 0.10. The land cover factor presented a partial eta-squared statistic (η_p_^2^) of 56.21%, while a much lower value of 17.18% was attained for the stereo pair factor. In the case of NMAD, both factors resulted in being significant at *p* < 0.05 level, with η_p_^2^ values of 41.73% and 40.06% for land cover and stereo pairs, respectively. Overall, the best vertical accuracy results, considering all statistic descriptors used, were achieved in the case of bare soil, followed by plastic covered greenhouse and, finally, urban areas. Regarding the sensor tested, the vertical accuracies from Deimos-2 stereo pairs were worse than the ones derived from WV2.

Focusing on the 9 DSMs corresponding to the plastic covered greenhouse land cover, significant differences at *p* < 0.05 (for SD and LE95) and *p* < 0.10 (for RMSE_Z_) were found between the WV2 stereo pair and the Deimos-2 stereo pair acquired in July. However, no significant differences were detected when comparing the DSMs derived from the WV2 stereo pair and the second stereo pair from Deimos-2 (August). More robust significant differences were achieved between the two Deimos-2 stereo pairs and the WV2 one when using NMAD.

With regard to urban areas, significant differences (*p* < 0.05) were detected for SD, RMSE_Z_ and LE95 statistics between the WV2-based DSM and the DSM derived from the stereo pair of Deimos-2 taken in August, statistically significant differences were not observed with respect to the DSM based on the stereo pair of Deimos-2 acquired in July. Again, NMAD was able to clearly differentiate the worst results obtained with the stereo pairs of Deimos-2.

Finally, no significant differences could be found using standard statistics (SD, RMSE_Z_ and LE95) between the DSMs produced from WV2 and Deimos-2 stereo pairs when working on bare soil land cover, mainly due to the variability in the vertical accuracy attained for the Deimos-2 imagery (Table 6). However, NMAD did detect significant differences (*p* < 0.05), indicating that the vertical accuracies obtained from WV2 were significantly better than those obtained with Deimos-2.

## 6. Discussion

### 6.1. Sensor Orientation of Deimos-2 Images

The RMSE_2D_ values attained from the direct geopositioning approach were always lower than the nominal circular error 90% (CE90) of 100 m (equivalent to approximately 65.9 m as RMSE_2D_) specified for Deimos-2 L1B products without GCPs’ support [16]. However, these direct geopositioning errors provided by Deimos-2 were almost four times higher than those obtained for the Maxar’s (old DigitalGlobe) VHR satellite constellation, where the CE90 for WV2 ORS2A product turns out to be 10.2 m (6.7 m in terms of RMSE_2D_) [46]. It is worth noting that this achievement is not really important in practical terms because the sensor orientation phase when working on VHR satellite images is usually supported by accurate GCPs [1,6,7].

As already mentioned, a zero-order polynomial adjustment (RPC0) is adequate for most of the modern VHR satellites when using the triangulation method developed by Grodecki and Dial [12]. However, according to Fraser and Hanley [13], when there are high order distortions in the images, adding terms to the image-to-space transformation by using RPC1 or RPC2 instead of RPC0 is required. In the light of these findings, Deimos-2 L1B single images showed important distortions that required applying the RPC1 sensor model and 12 GCPs for achieving the best orientation results.

Fraser and Ravanbakhsh [4] reported that the planimetric accuracy at the sensor orientation phase should be in the range of 0.5–0.7 pixels for VHR satellites when using refined RPCs supported with a few high-accuracy GCPs. In this way, the attained geometric accuracy for Deimos-2 images using RPC1 and 12 GCPs (RMSE_2D_ value of 1.225 m or 1.225 pixels) can be considered as suboptimal results. These poor results were influenced by their very large off-nadir viewing angle (ranging from 34.32° to 36.45°). Note that Aguilar et al. [7] reported RMSE_2D_ values of 0.89 pixels and 1.10 pixels in the orientation phase of two WV2 ORS2A single images with an off-nadir angle of 10° and 22.4°, respectively. In addition, and according to Vajsová et al. [16], the corresponding ground spatial resolution of Deimos-2 images with an off-nadir angle of 35° would be 1.9 m. In this way, the update of the GSD could improve the expected RMSE_2D_ to 0.66 pixels (1.255/1.9).

### 6.2. Quality Assessment of the Extracted DSMs

Regarding completeness, an interesting comparison can be made between Qin’s RSP software [23] and OrthoEngine v. 2018 in the case of the WV2 stereo pair. The completeness values, in the same subareas and using the same methodology as in the current work, were reported for the WV2 data by Aguilar et al. [19]. Both software packages used a SGM matching algorithm, but slightly better completeness values were provided by RSP in each land cover. The completeness values attained using OrthoEngine in this work (Table 5) were 92.00%, 86.04% and 99.76% for plastic covered greenhouse, urban, and bare soil, respectively, while values of 97.92% (greenhouse), 94.83% (urban) and 99.97% (bare soil) were reported by Aguilar et al. [19] using RSP. These results are consistent with those reported by Han et al. [21]. Moreover, the SGM approach used in this work achieved much better completeness values than other algorithms such as FORSAT [21] or the algorithm included in OrthEngine v. 2013 (it is also available in OrthoEngine v. 2018) based on the cross-correlation area-based matching procedure [19].

The vertical accuracy results of the DMS produced from WV2 data were always better than those computed on the DSMs derived from Deimos-2 imagery for all the three land covers tested. This was mainly because of the higher spatial resolution of PAN WV2 imagery (0.5 m GSD) as compared to Deimos-2 (1 m GSD). If the RMSE_Z_ values for plastic covered greenhouse areas are presented in relation to their corresponding sensor ground sample distance (i.e., RMSE_Z_/sensor GSD), vertical errors of 1.94, 1.76 and 1.48 times the GSD are attained for WV2, Deimos-2 July, and Deimos-2 August, respectively. If the same exercise is applied to bare soil land cover, values of 1.43 for WV2, 1.32 for Deimos-2 July, and 1.41 for Deimos-2 August are obtained. Finally, RMSE_Z_ values of 4.41 times the GSD for WV2, 3.55 for Deimos-2 July, and 3.96 for Deimos-2 August are achieved in urban areas. All these values are in accordance with what is reported in the literature for those different land covers [19].

The use of the robust scale estimator NMAD yielded clearer significant differences in the statistical analysis. According to Höhle and Höhle [44], the removal of outliers by a threshold (e.g., three-sigma rule) could not eliminate all of the outliers. In this sense, these kind of robust accuracy measures can help to improve the understanding of DEM errors. Therefore, they should be integrated in DSM validation reports [47]. In our work, all the SD values were quite a lot higher than NMAD even though a small percentage of outliers ranging from 1% to 3% was detected.

Finally, if we compare the SD values computed on the DSM produced from the WV2 stereo pair by using OrthoEngine (Table 3) and RSP software (see Aguilar et al. [19]), worse vertical accuracies are always attained from RSP software. Summing up, 0.86 m for OrthoEngine against 1.10 m for RSP in plastic covered greenhouse areas, 0.21 m for OrthoEngine against 0.28 m for RSP in bare soil areas, and 2.16 m for OrthoEngine against 2.92 m for RSP in urban areas. This trend can also be seen in other land covers such as impervious objects, industrial buildings, residential buildings, and roads [21].

## 7. Conclusions

This paper is focused on the capabilities of Deimos-2 PAN L1B stereo pairs (1 m GSD) to extract high quality DSMs over different land covers. A statistical analysis of the sensor orientation phase for Deimos-2 imagery is carried out as a previous step. The statistical analysis of the obtained results recommends the use of the RPC1 model with 12 GCPs to carry out the orientation phase of the Deimos-2 optical sensor.

Concerning the extracted DSMs, the best quality results for both completeness and vertical accuracy were achieved from the WV2 stereo pair used as benchmark, mainly due to its lower GSD. However, the quality of the DSMs attained from Deimos-2 stereo pairs also yielded very good figures. The major differences between WV2 and Deimos-2 were observed in very uneven urban land cover areas, where the lower GSD of WV2 imagery produced DSMs with higher vertical accuracy. In that sense, the SGM method applied to extract DSMs could have minimized the differences attributable to the optical sensor, especially in urban environments, where SGM usually achieves its better performance.

Finally, it should be emphasized that the results obtained on the quality of the DSMs produced from Deimos-2 could be classified as good or bad according to the level of detail required for their application. It is important to note that, in the frame of the research project about plastic covered greenhouse detection in which our team is working on, SD values ranging from 1.07 m to 1.78 m turn out to be too high for the objectives pursued.

## Figures and Tables

**Table 1:** Characteristics of panchromatic (PAN) images from Deimos-2 acquired at the study site.

Product	Deimos-2 Stereo Pair		Deimos-2 Stereo Pair	
Acquisition date	30 July 2019	30 July 2019	5 August 2019	5 August 2019
Image Code	1_1	1_2	2_1	2_2
Acquisition time (GTM)	11:01:58	11:03:43	11:00:11	11:01:56
Cloud cover	0%	0%	0%	0%
Sun elevation	65.438	65.668	64.068	64.278
Sun azimuth	132.908	133.718	134.438	135.238
Collection elevation	54.758	53.548	55.678	54.698
Collection azimuth	192.868	324.828	187.378	329.468
Product pixel size	1 m	1 m	1 m	1 m

**Table 2:** Direct geopositioning accuracy at 102 ICPs (without GCPs) given as bias, standard deviation (SD) and RMSE for the four single Deimos-2 images.

Image Code	Bias (m)		SD (m)			RMSE (m)		
	X	Y	X	Y	2D	X	Y	2D
1_1	11.12	−22.10	0.97	0.82	1.27	11.16	22.12	24.77
1_2	11.86	23.22	1.11	1.04	1.52	11.91	23.25	26.12
2_1	7.34	−21.65	0.86	1.07	1.37	7.38	21.67	22.90
2_2	9.20	23.05	0.91	1.14	1.46	9.24	23.08	24.86

**Table 3:** Sensor orientation accuracy (RMSE) using rational polynomial coefficient RPC0, RPC1 and RPC2 sensor models supported by 102 GCPs.

Image Code	Sensor Model	RMSE (m)		
		X	Y	2D
1_1	RPC0	0.967	0.812	1.262
	RPC1	0.770	0.650	1.008
	RPC2	0.754	0.645	0.993
1_2	RPC0	1.106	1.037	1.516
	RPC1	0.869	0.842	1.210
	RPC2	0.854	0.814	1.180
2_1	RPC0	0.857	1.062	1.365
	RPC1	0.803	0.669	1.045
	RPC2	0.745	0.623	0.971
2_2	RPC0	0.902	1.138	1.452
	RPC1	0.802	0.730	1.085
	RPC2	0.756	0.678	1.016

**Table 4:** Comparison of mean values of RMSE_2D_ computed at ICPs from Deimos-2 PAN images depending on the number of GCPs (no. GCPs). For each sensor model tested, mean values of RMSE_2D_ in brackets followed by different superscript letters indicate significant differences at a significance level *p* < 0.05.

Sensor Model (RMSE_2D_ (m))	No. GCPs	ICPs	RMSE_2D_ (m) (Mean Value of the Four Deimos-2 Images)
**RPC0** (1.453 ^a^)	6	96	1.417
	12	90	1.477
	18	84	1.467
**RPC1** (1.268 ^b^)	6	96	1.317
	12	90	1.255
	18	84	1.231
**RPC2** (1.234 ^b^)	6	96	1.301
	12	90	1.213
	18	84	1.189

**Table 5:** Mean and range of completeness values (maximum and minimum) attained from the three samples per land cover. For each land cover, different superscript letters between data along the completeness column indicate significant differences at a significance level *p* < 0.05 (* indicates differences at a significance level *p* < 0.10).

Land Cover	Stereo Pair	Completeness (%)	Min. (%)–Max. (%)
Plastic covered greenhouse	Deimos-2 (30 July 2019)	90.98	83.61–95.62
	Deimos-2 (5 August 2019)	91.88	89.31–94.11
	WV2 (5 July 2015)	92.00	89.10–93.75
Urban	Deimos-2 (30 July 2019)	79.14 ^a,b^	76.50–80.76
	Deimos-2 (5 August 2019)	71.01 ^a^	65.96–78.92
	WV2 (5 July 2015)	86.04 ^b^	84.39–87.00
Bare Soil	Deimos-2 (30 July 2019)	95.96 ^a,b,^*	94.02–98.09
	Deimos-2 (5 August 2019)	92.73 ^a,^*	85.38–98.58
	WV2 (5 July 2015)	99.76 ^b,^*	99.47–99.95

**Table 6:** Vertical accuracy (mean, SD, RMSE_Z_, 95th percentile linear error (LE95) and normalized median absolute deviation (NMAD)) computed at LiDAR reference ICPs. All the depicted values are mean values corresponding to three samples for each land cover. Minimum and maximum values for the three samples are depicted in brackets and italic font for mean and SD. For each land cover, different superscript letters between data along the same column indicate significant differences at a significance level *p* < 0.05 (* indicates differences at a significance level *p* < 0.10).

Land Cover	Stereo Pair	Mean (m)	SD (m)	RMSE_Z_ (m)	LE95 (m)	NMAD (m)
Plastic covered greenhouse	Deimos-2 (30 July 2019)	0.52 (*1.60*, −*0.58*)	1.44 ^a^ (*1.78*, *1.21*)	1.76 ^a,^*	3.59 ^a^	1.02 ^a^
	Deimos-2 (5 August 2019)	0.30 (*1.60*, −*0.43*)	1.19 ^a,b^ (*1.31*, *1.07*)	1.48 ^a,b,^*	2.93 ^ab^	0.92 ^a^
	WV2 (5 July 2015)	0.43 (*0.62*, *0.19*)	0.86 ^b^ (*0.65*, *1.11*)	0.97 ^b,^*	2.10 ^b^	0.45 ^b^
Urban	Deimos-2 (30 July 2019)	0.04 (*2.24*, −*1.71*)	3.16 ^a,b^ *(3.63*, *2.91*)	3.55 ^a,b^	7.50 ^a,b^	2.14 ^a^
	Deimos-2 (5 August 2019)	−0.14 (*0.43*, −*0.63*)	3.93 ^a^ (*4.69*, *3.02*)	3.96 ^a^	8.62 ^a^	2.92 ^a^
	WV2 (5 July 2015)	0.37 (*0.49*, *0.30*)	2.16 ^b^ (*3.05*, *1.59*)	2.20 ^b^	5.38 ^b^	0.75 ^b^
Bare Soil	Deimos-2 (30 July 2019)	0.04 (*0.76*, −*1.09*)	1.06 (*1.71*, *0.60*)	1.32	2.99	0.81 ^a^
	Deimos-2 (5 August 2019)	−0.27 (*0.97*, −*1.42*)	1.06 (*1.99*, *0.42*)	1.41	3.20	0.73 ^a,b^
	WV2 (5 July 2015)	0.68 (*0.79*, *0.61*)	0.21 (*0.24*, *0.19*)	0.71	1.03	0.17 ^b^
